# Amplitude-Integrated Electroencephalography and Brain Oxygenation for Postcardiac Arrest Patients with Targeted Temperature Management

**DOI:** 10.1089/ther.2018.0051

**Published:** 2019-09-10

**Authors:** Shingo Ihara, Atsushi Sakurai, Kosaku Kinoshita, Junko Yamaguchi, Atsunori Sugita

**Affiliations:** Division of Emergency and Critical Care Medicine, Department of Acute Medicine, Nihon University School of Medicine, Tokyo, Japan.

**Keywords:** aEEG, rSO_2_, out-of-hospital cardiac arrest, targeted temperature management, outcome

## Abstract

Brain injury is the most common cause of death postcardiac arrest. Amplitude-integrated electroencephalography (aEEG) is suggested to be useful in the prognostication in cases of postcardiac arrest brain injury. However, combined monitoring with aEEG and regional oxygen saturation (rSO_2_) for postcardiac arrest syndrome (PCAS) patients to improve accuracy has not been reported. The purpose of this prospective observational study is to assess the usefulness of aEEG and rSO_2_ for PCAS patients with targeted temperature management (TTM) to predict neurological outcome and possibly identify the pathophysiology of postcardiac arrest brain injury. PCAS patients with TTM at 34°C were monitored by aEEG and rSO_2_ immediately after admission to the intensive care unit and evaluated at the start of monitoring, and 24 and 48 hours after return of spontaneous circulation (ROSC). Patients were divided into two groups according to electroencephalography (EEG) pattern: a continuous EEG (C) pattern group and a noncontinuous EEG (NC) pattern group. Patients with C pattern had a significantly more favorable neurologic outcome compared with patients with an NC pattern at each point in time. No significant difference in rSO_2_ values was observed between the C pattern and the NC pattern at any time point. Variation coefficient at rSO_2_ in the NC group was significantly greater than that in the C group from the start of the monitoring to 24 hours. aEEG is useful in predicting outcome for PCAS patients whereas rSO_2_ is not.

## Introduction

Despite recent medical advances, the outcome of patients resuscitated from cardiac arrest is still very poor. In Japan, 87% of patients experiencing cardiac arrest outside of the hospital died and even in cases of witnessed arrest and with cardiac etiology, only 9% achieved a good neurological outcome (SOS-KANTO 2012 Study Group, 2015). The International Liaison Committee on Resuscitation defined causes of postcardiac arrest syndrome (PCAS) into four types of pathophysiology: postcardiac arrest brain injury, postcardiac arrest myocardial dysfunction, systemic ischemia/reperfusion response, and persistent precipitating pathology (Nolan *et al.*, 2008). Among these pathophysiology types, postcardiac arrest brain injury is the most common cause of death (Dragancea *et al.*, 2013), and it greatly disrupts rehabilitation in cases of PCAS patients who survive. Therefore, investigation of brain injury for PCAS patients can be considered one of the most important and crucial approaches to improve outcome. Usually, PCAS patients are treated with targeted temperature management (TTM) in the intensive care unit (ICU) (Callaway *et al.*, 2015). In this situation in the ICU, several kinds of neuro-monitoring modalities, such as electrophysiology, intracranial pressure measurements, and brain oxygenation, are suggested to be useful in the prognostication and illustration of the pathophysiology in cases of postcardiac arrest brain injury (Reis *et al.*, 2017).

Rundgren *et al.* (2006, 2010) reported that amplitude-integrated electroencephalography (aEEG) is useful to predict neurological outcomes for PCAS patients with TTM. In these studies, aEEG measured with PCAS patients was divided into the following four patterns: continuous, flat, burst suppression (BS), and electrographic status epilepticus (ESE). Patients with continuous pattern tended to have a favorable neurological outcome whereas those with BS or ESE tended to have an unfavorable neurological outcome. Neurological outcome of initial flat patterns was difficult to predict, because some flat patterns changed to continuous patterns during TTM and became favorable neurological outcomes. These facts indicated that the electrophysiological situation in postcardiac arrest brain injury was associated with the severity of brain damage and might change over time.

Brain oxygenation status between circulation and metabolism can be measured by regional oxygen saturation (rSO_2_) using the near-infrared spectroscopy (NIRS) method. NIRS is a noninvasive method used to monitor PCAS patients. Three studies reported that regional cerebral oxygenation of PCAS patients with good outcome was significantly better compared with those with poor outcome (Meex *et al.*, 2013; Ahn *et al.*, 2014; Storm *et al.*, 2014), although one study did not show any such association (Ibrahim *et al.*, 2015). Further investigation to clarify the pathophysiology of postcardiac arrest brain injury, through monitoring by rSO_2_ at the ICU, is needed.

One study reported rScO_2_ and aEEG measurements for infants with perinatal asphyxia (Lemmers *et al.*, 2013). However, combined monitoring with aEEG and rSO_2_ for adult PCAS patients with TTM has not been reported. The aim of this study was to assess aEEG and rSO_2_ of PCAS patients with TTM, which may be useful in predicting neurological outcome and may illustrate the pathophysiology of postcardiac arrest brain injury.

## Methods

### Patients

This prospective observational study was performed at the ICU at Nihon University Itabashi Hospital. Approval was obtained from the Clinical Research Institutional Review Board (IRB) of Nihon University School of Medicine Itabashi Hospital (RK-121109-1). This study included comatose survivors of out-of-hospital PCAS patients aged 20 years or older who were treated with TTM from July 1, 2012 to June 31, 2015. This study included consecutive patients with TTM, but patients were excluded in cases of (1) death within 72 hours after cardiac arrest, (2) history of neurodegenerative disease, or (3) severe traumatic brain injury. Among patients who received TTM, patient characteristics of age, gender, cause of cardiac arrest, witness, shockable rhythm, bystander cardiopulmonary resuscitation (CPR), and mortality between included and excluded patients in this study were compared.

### TTM protocol

Patients who remained comatose after return of spontaneous circulation (ROSC) were treated with TTM at 34°C for 24 hours. Exclusion criteria of TTM included hemodynamic instability refractory to the use of vasopressor agents, respiratory failure (PaO_2_/FiO_2_ <200), and terminal illness before cardiac arrest. After ROSC, patients were sedated (midazolam, 0.08 mg/kg intravenously) and paralyzed (rocuronium, 0.8 mg/kg intravenously) for shivering control, followed by continuous infusion of midazolam (0.05–0.1 mg/kg/h), fentanyl (1 μg/kg/h), and rocuronium (0.3–0.6 mg/kg/h). Hypothermia was induced by cold saline (4°C 30 mL/kg) and a surface cooling device (Arctic Sun system, Medivance, Louisville, CO) for the maintenance of TTM. Thereafter, patients were rewarmed at a maximum of 0.1°C/h to normothermia. Targeted management parameters were as follows: mean blood pressure (BP) >65 mmHg, SpO_2_ >94% to <97%, PaCO_2_ >35 to <45 mmHg, and hemoglobin (Hb) >7 g/dL.

### aEEG and rSO_2_ monitoring and analysis

All patients were monitored with an aEEG (NicoletOne; Cardinal Health, Dublin, OH) and rSO_2_ (INVOS 5100 C; Covidien, Boulder, CO) as soon as possible after arrival at the ICU. The aEEG was examined by using six subdermal electrodes in the left frontal (F3), right frontal (F4), left parietal (P3), and right parietal (P4) positions with ground in the frontal midline and reference in the central midline. The NIRS probes were placed on the left side forehead of each patient to detect frontal cerebral oxygen saturation. aEEG patterns were classified into four categories: continuous, flat, BS, and ESE, as previously described by Rundgren (Rundgren *et al.*, 2006, 2010). aEEG, rSO_2_, and parameters (mean BP, SpO_2_, PaCO_2_, Hb, and body temperature) were evaluated at the start of the monitoring, and 24 and 48 hours after ROSC. Patients were divided into two groups according to electroencephalography (EEG): a continuous EEG (C) pattern group and a noncontinuous EEG (NC) pattern group. The C pattern group was determined by a continuous aEEG pattern, and the NC pattern group was determined by a flat, BS, or ESE aEEG pattern.

### Outcome assessment

Neurological outcome was assessed by using the Cerebral Performance Category (CPC) scale by the ICU physician at the time of discharge from Nihon University School of Medicine Itabashi Hospital (Cummins *et al.*, 1991). Favorable neurologic outcome was defined as a CPC score of 1 or 2, and unfavorable outcome was defined as a CPC score of 3–5.

### Statistical analysis

Statistical analysis was performed by using SPSS (IBM SPSS Version22). Fisher's exact probability test, *t*-test, and Mann-Whitney *U* test were used to determine statistical significance. Range of variation within rSO_2_ was evaluated by the coefficient of variation [CV: (standard deviation/mean) × 100]. Significant difference at CV between the two groups was calculated by the likelihood ratio test based on the hypothesis that k normally distributed populations share the same CV (Verrill and Johnson, 2007). Probability values of *p* < 0.05 were considered statistically significant.

## Results

During the study period, 1266 out-of-hospital cardiac arrest (OHCA) patients were admitted to the hospital and 375 of these had ROSC. One hundred and fifty patients received TTM, and 49 patients were monitored by using aEEG and rSO_2_. One hundred and one patients were excluded due to lack of measurement instruments (*n* = 79), hemodynamic instability during TTM (18), consent not obtained (3), and operation for pulmonary embolism (1). Twenty-one patients did not improve consciousness and died. There was no patient withdrawal. Table 1 shows patient characteristics. The age of all patients was 64.7 ± 16.0 (mean ± standard deviation [SD]) years old, and monitoring was initiated at a median time of 351 ± 182 minutes after ROSC. Comparing the two groups, the ratio of men was significantly larger and the time interval from arrest to ROSC was significantly smaller in the C pattern group than in the NC pattern group. Characteristics of the 80 excluded patients with TTM (lack of measurement instruments and operation for pulmonary embolism) were: mean age 62.0 years, ratio of males was 47.5%, cardiac cause 56.2%, witness 60.0%, shockable rhythm 31.3%, bystander CPR 53.8%, and favorable neurological outcome 31.3%. Regarding characteristics of the 49 included patients and those of the 80 excluded patients with TTM, no statistically significant difference was observed.

**Table 1.** Comparison of Patient Characteristics Between Patients with C and NC Patterns

	*All patients (*n* = 49)*	*C pattern (*n* = 13)*	*NC pattern (*n* = 36)*	p
Age, years, mean ± SD	64.7 ± 16.0	64.0 ± 13.9	64.9 ± 16.9	0.642
Male, *n* (%)	29 (59.2)	11 (84.6)	18 (50.0)	0.047
Cardiac cause, *n* (%)	28 (57.1)	9 (69.2)	19 (52.8)	0.348
Witnessed, *n* (%)	37 (75.5)	11(84.6)	26 (72.2)	0.474
Shockable rhythm, *n* (%)	18 (36.7)	6 (46.2)	12 (33.3)	0.325
Bystander CPR, *n* (%)	25 (51.0)	10 (76.9)	15 (41.7)	0.051
Time from arrest to ROSC, minute, mean ± SD	31.7 ± 19.7	15.6 ± 14.8	37.4 ± 18.1	0.001
Time from ROSC to aEEG and rSO_2_ application, minute, mean ± SD	351.0 ± 182.4	365.8 ± 192.0	345.6 ± 181.3	0.667

aEEG, amplitude-integrated electroencephalography; C pattern, continuous EEG pattern; CPR, cardiopulmonary resuscitation; NC pattern, noncontinuous EEG pattern; ROSC, return of spontaneous circulation; rSO_2_, regional oxygen saturation; SD, standard deviation.

No significant difference between the parameters of the C pattern group and the NC pattern group was observed, except for Hb at the start of the measurement (C vs. NC; 14.0 ± 1.7 g/dL vs. 12.4 ± 3.3 g/dL, *p* = 0.041) and after 48 hours (13.3 ± 3.0 g/dL vs. 10.8 ± 2.4 g/dL; *p* = 0.020), and mean BP after 24 hours (99.8 ± 17.9 mmHg vs. 86.8 ± 18.9 mmHg, *p* = 0.019). Similar results were found when parameters of patients with C patterns were compared with flat patterns.

**FIG. 1.**
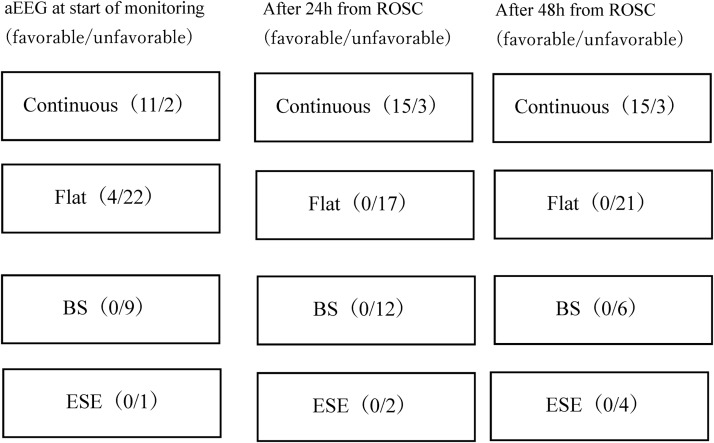
aEEG from the start of the monitoring to after 48 hours from ROSC monitoring (favorable/unfavorable). aEEG, amplitude-integrated electroencephalography; ROSC, return of spontaneous circulation.

**FIG. 2.**
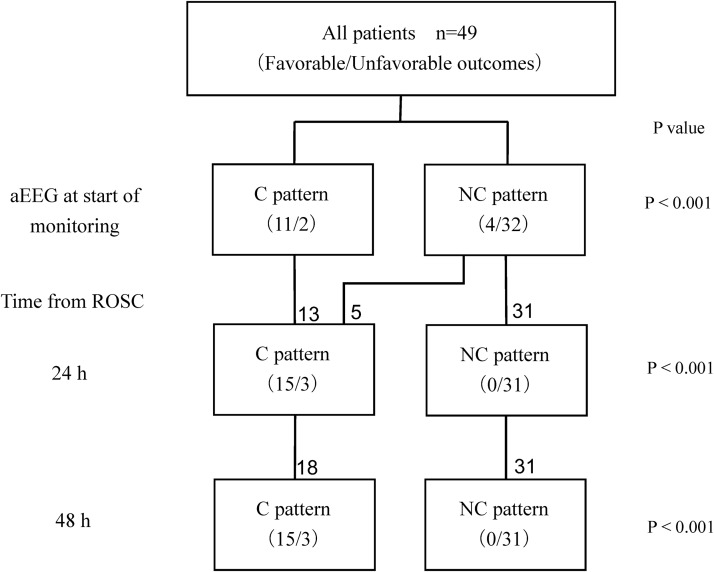
The C pattern group had a significantly more favorable neurologic outcome compared with the NC pattern group for all points in time. C group, continuous EEG group; NC group, noncontinuous EEG group.

At the start of the monitoring, the C pattern group was observed in 13 out of 49 patients, 11 of whom had favorable neurologic outcomes. The NC pattern group was observed in 36 patients (flat 26, BS 9, ESE 1), 4 of whom had favorable neurologic outcomes. At 24 hours after ROSC, the C pattern group was seen in 18 of 49 patients, 15 of whom had favorable neurologic outcomes. Thirty-one patients of the NC pattern group (flat 17, BS 12, ESE 2) did not have favorable neurologic outcomes. These results were similar to those at 24 hours after ROSC and at 48 hours (Fig. 1). Significantly favorable neurologic outcomes were observed for the C pattern group compared with the NC pattern group at all times (Fig. 2). At the start of the monitoring, the C pattern group showed sensitivity at 73.3% (95% confidence interval [CI], 55.1–82.4), specificity 94.1% (95% CI, 86.1–98.1), positive predictive value 84.6% (95% CI, 63.6–95.1), negative predictive value 88.9% (95% CI, 81.3–92.7), and odd ratio 44.0 (95% CI, 7.6–246.3) for favorable neurologic outcomes. Similar results were obtained after 24 and 48 hours with sensitivity 100% (95% CI, 84.8–100), specificity 91.2% (95% CI, 84.5–91.2), positive predictive value 83.3% (95% CI, 70.7–83.3), and negative predictive value 100% (95% CI, 92.7–100) (Table 2). C pattern was observed in all patients with favorable neurologic outcomes by 24 hours from ROSC.

**Table 2.** C Group Validity for Favorable Neurological Outcomes at Each Time Point

	*Sensitivity % (95% CI)*	*Specificity % (95% CI)*	*Positive predictive value (95% CI)*	*Negative predictive value (95% CI)*	*Odds ratio*
At start of monitoring C pattern group	73.3 (55.1–82.4)	94.1 (86.1–98.1)	84.6 (63.6–95.1)	88.9 (81.3–92.7)	44.0
After 24 h and 48 h from ROSC C pattern group	100 (84.8–100)	91.2 (84.5–91.2)	83.3 (70.7–83.3)	100 (92.7–100)	—

C group, continuous EEG pattern; CI, confidence interval.

**FIG. 3.**
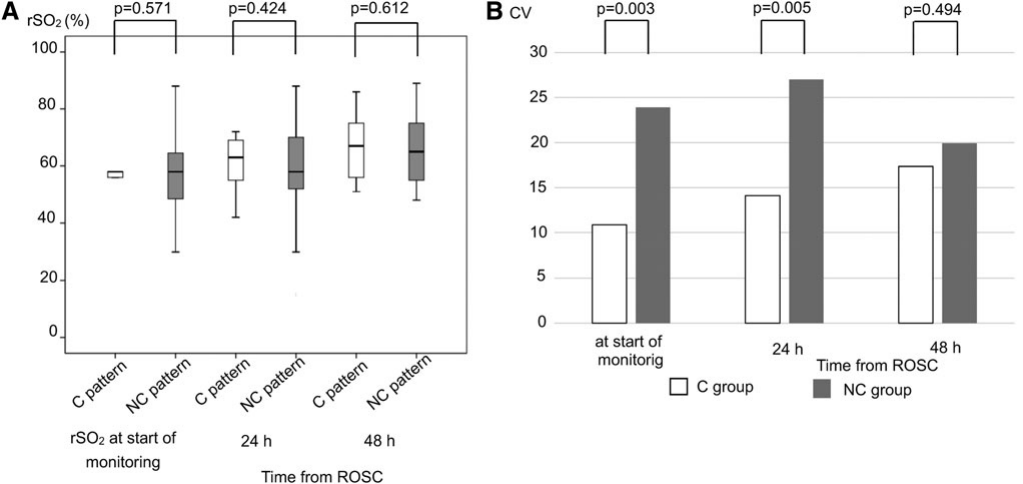
**(A)** No significant differences in mean rSO_2_ values between the C and NC groups were observed at each point in time. **(B)** CV = standard deviation/mean × 100. CV for rSO_2_ in the NC group was significantly greater than that in the C group at the start of the monitoring and 24 hours after ROSC. CV, coefficients of variation; rSO_2_, regional oxygen saturation.

No significant difference in rSO_2_ values was observed between the C pattern group and the NC pattern group at all points in time (Fig. 3a). CV at rSO_2_ in the NC group was significantly greater than that in the C group at the start of the monitoring and 24 hours after ROSC (Fig. 3b). Regarding CV, no significant differences in parameters between C pattern and NC pattern groups were observed, except Hb at the start of measuring (C vs. NC: 12.4 vs. 26.6, *p* = 0.0029).

Five patients who initially had a flat pattern changed to the C pattern group and 21 patients remained in the NC pattern group at 24 and 48 hours after ROSC. Four out of five cases of C patterns showed favorable neurological outcomes at 24 and 48 hours after ROSC. All 21 patients with an NC pattern had an unfavorable neurological outcome at 24 and 48 hours after ROSC. The rSO_2_ values between the two groups were compared, and no significance difference at any point in time was observed at the start of the measurement (flat to C pattern vs. flat to NC pattern; 54.3% ± 6.2% vs. 57.9% ± 12.5%, *p* = 0.408), after 24 hours (61.5% ± 12.6% vs. 58.3% ± 9.7%, *p* = 0.408) and after 48 hours (69.0% ± 14.1% vs. 60.3% ± 11.3%, *p* = 0.245).

## Discussion

An initial C pattern or an evolution into C pattern was strongly associated with favorable neurological outcomes. An BS pattern or ESE at any time was associated with unfavorable neurological outcomes. There were no differences in rSO_2_ between the C pattern group and the NC pattern group at any time point.

In this study, patients with a C pattern on aEEG from an early stage had a favorable neurological outcome. Time interval from arrest to ROSC was significantly shorter for the C pattern group than that for the NC pattern group. This time interval indicates brain ischemic time and intensity of brain injury (Komatsu *et al.*, 2014). These facts suggested that in cases where the C pattern could be observed at the early phase after ROSC in PCAS patients, postcardiac arrest brain injury would be mild. However, outcome for patients with a flat pattern within 24 hours after ROSC was difficult to predict, as the pattern may change other patterns over time. Rundgren *et al.* (2010) classified the aEEG waveform into 4 patterns and reported that the 29 out of 32 patients with an initial waveform of continuous pattern had a favorable neurological outcome. In this study, to more conveniently evaluate the aEEG pattern, it was classified into two types, C pattern and NC pattern. aEEG monitoring started ∼6 hours from ROSC on average. When aEEG at that time was C pattern, favorable neurological outcome could be predicted with an accuracy of positive predictive value of 84.6%. Moreover, unless a patient showed a C pattern within 24 hours after ROSC, the patient had an unfavorable outcome. aEEG is useful in predicting outcome for PCAS patients, and accuracy of outcome prediction is improved by measuring aEEG over time.

The NC group is composed of flat, BS, and ESE patterns. BS and ESE were reported as unfavorable outcomes. Bassetti *et al.* (1996) reported the Hockaday classification, which are EEG classifications of cases after cardiac arrest. Among this classification, BS was classified as Grade IV and was reported to be associated with an unfavorable outcome (Bassetti *et al.*, 1996). In this study, patients with BS at any time points had an unfavorable outcome. Therefore, brain injury in patients with BS pattern is considered severe.

The ESE pattern was reported to be associated with unfavorable outcomes (Rossetti *et al.*, 2007). In this study, patients with ESE at any time point had unfavorable outcomes. However, even in the case of ESE pattern, patients who follow the course of favorable outcomes have been reported (Hovland *et al.*, 2006; Sunde *et al.*, 2006). In reports using aEEG, ESE was reported to be divided into two types (Friberg *et al.*, 2013): a type that changes from a BS pattern to an ESE pattern during the early stage of TTM, and a type that changes from a continuous to an ESE pattern during rewarming to postrewarming. The former type led to an unfavorable outcome, but the latter type may have a favorable outcome. When evaluating ESE, it is important to confirm background EEG and the appearance time of the ESE pattern.

Referring to a study by Storm *et al.* (2014), in patients with unfavorable outcomes in the early phase after resuscitation from OHCA, a large variation in SD of rSO_2_ was observed. In a study on cerebral oxygen saturation in hypoxic ischemic encephalopathy in newborns, the rSO_2_ value of patients with unfavorable neurological outcomes was wide at the early point in time after birth (Lemmers *et al.*, 2013). However, the study did not consider whether there was a large variation in SD of rSO_2_ in patients with unfavorable outcomes. This study also showed that CV of rSO_2_ in the NC group was significantly greater than that in the C group, although there was no significant difference between C and NC patterns at any point in time. Hence, from this fact, the degree of variation in rSO_2_ values in the NC group changed more than in the C group in the early phase after ROSC. As previously mentioned, brain damage in most patients in the C group must be mild. These facts suggest that if brain damage is severe, the value of rSO_2_ may widely vary in patients with whole-brain ischemia in the early phase after reperfusion.

Since rSO_2_ and jugular bulb venous oxygen saturation are correlated (Kim *et al.*, 2000), factors of varying rSO_2_ are represented by the following formula:

\begin{align*}
{ \rm{rS}}{{ \rm{O}}_{ \rm{2}}}{ \rm{ \; = \;Sa}}{{ \rm{O}}_{ \rm{2}}}{ \rm{ - CMR}}{{ \rm{O}}_{ \rm{2}}}{ \rm{ / }} \left( {{ \rm{CBF \; \times \;1}}{ \rm{.34 \; \times \;Hb}}} \right).
\end{align*}

cerebral blood flow (CBF) is affected by BP, PaCO_2_. Parameters of related rSO_2_ did not vary, except for Hb at the start of the measurement. Therefore, variation of Hb at the start of the monitoring may affect CV of rSO_2_.

In the normal brain, pressure autoregulation keeps CBF constant (Kinoshita, 2016). Therefore, in an uninjured brain, even if BP changes within a certain range, change in rSO_2_ value is hardly observed. However, in a past report on CBF in PCAS patients, the pressure autoregulation mechanism was impaired (Sundgreen *et al.*, 2001). Pressure autoregulation in PCAS patients either shifted to the right or was linearly correlated with BP. According to this report, pressure autoregulation in PCAS patients with unfavorable neurological outcomes was impaired and various values of CBF could be observed as BP changes. Another report showed that pressure autoregulation in PCAS patients with unfavorable outcomes was impaired, using NIRSs (Ameloot *et al.*, 2015). In patients with unfavorable neurological outcomes, cerebral oxygen saturation varied along with changes in BP; whereas in patients with favorable outcomes, cerebral oxygen saturation was constant even if BP changed.

In this study, this phenomenon seemed to continue from the start of the measurement to 24 hours, and other studies also showed that this continued at 12 hours (Storm *et al.*, 2014) or 24 hours (Lemmers *et al.*, 2013). Ehara *et al.* (2017) reported that in cardiac arrest patients with CPR, rSO_2_ values significantly increased just after starting extracorporeal cardiopulmonary resuscitation (ECPR) in the poor neurological group. On the other hand, cerebral rSO_2_ values did not increase significantly during ECPR in the good neurological group. One explanation is that impairment of autoregulation in patients with severe brain damage may begin immediately after resuscitation by ECPR and CBF may increase by perfusion of ECPR, leading to an increased rSO_2_ value.

In this study, CV of rSO_2_ was not significantly different between C and NC groups 48 hours after ROSC. Pham *et al.* (2015) reported that 4 out of 14 patients with impaired autoregulation improved over 3 days after ROSC. Consequently, patients in the NC group with impaired autoregulation may improve 48 hours after ROSC. The cerebral oximetry index (COx) estimates the autoregulation of the brain by using mean arterial pressure and rSO_2_ (Ameloot *et al.*, 2015). COx is calculated as the linear correlation coefficient by using values of BP and cerebral oxygen saturation. When COx shows a positive correlation, it indicates impaired autoregulation of the brain. When COx shows a negative or near-zero correlation, autoregulation of the brain is maintained. Further study to evaluate autoregulation using COx is necessary.

Taken together, more accurate prediction of neurological outcomes by simultaneously monitoring aEEG and rSO_2_ is considered difficult due to the wide range of obtained rSO_2_ values in the NC pattern. This fact suggests that autoregulation may be impaired in cases of severe brain damage at the early stage of resuscitation in patients in the NC group.

### Limitation

Many studies on postcardiac arrest patients undergoing hypothermia have also been evaluated for prognosis after 3 to 6 months from ROSC. Because this study assesses neurological outcomes at hospital discharge, it is shorter than the general assessment period. However, there are reports that CPC at discharge and long-term prognosis on an annual basis are related (Hsu *et al.*, 2014). This article suggests that aEEG pattern, rSO_2_, and neurological outcome data in this study are informative in regards to prognostic prediction. Further study to examine the relationship between aEEG pattern, rSO_2_ value, and long-term outcomes is needed.

In our study, rSO_2_ was measured on the left side of the forehead only, compared with the Storm *et al.* study, which measured rSO_2_ average of both sides of the forehead. Toyama *et al.* (2015) reported no difference between right and left rSO_2_ values in the measurement of rSO_2_ by using INVOS during the carotid artery stenting operation. Therefore, we measured left-side rSO_2_ values only. However, whether there is a left**–**right difference of rSO_2_ values is not clear in PCAS patients, and further methodological investigation is required to clarify the difference in results from the Storm study.

## Conclusion

EEG is a useful tool that is used to predict the outcomes of PCAS patients. On the other hand, rSO_2_ values cannot be used to predict outcome due to the wide range in rSO_2_ values in PCAS patients with an abnormal aEEG pattern.
